# Model-Based Optimisation and Control Strategy for the Primary Drying Phase of a Lyophilisation Process

**DOI:** 10.3390/pharmaceutics12020181

**Published:** 2020-02-20

**Authors:** Brecht Vanbillemont, Niels Nicolaï, Laurens Leys, Thomas De Beer

**Affiliations:** 1 Laboratory of Pharmaceutical Process Analytical Technology (LPPAT), Department of Pharmaceutical Analysis, Faculty of Pharmaceutical Sciences, Ghent University, Ottergemsesteenweg 460, 9000 Ghent, Belgium; Brecht.Vanbillemont@UGent.be (B.V.); Laurens.Leys@UGent.be (L.L.); 2 BIOMATH, Department of Data Analysis and Mathematical Modelling, Faculty of Bioscience Engineering, Ghent University, Coupure Links 653, 9000 Ghent, Belgium; Niels.Nicolai@gci.ulaval.ca

**Keywords:** freeze-drying, supervisory process control, primary drying, dynamic design space, uncertainty analysis

## Abstract

The standard operation of a batch freeze-dryer is protocol driven. All freeze-drying phases (i.e., freezing, primary and secondary drying) are programmed sequentially at fixed time points and within each phase critical process parameters (CPPs) are typically kept constant or linearly interpolated between two setpoints. This way of operating batch freeze-dryers is shown to be time consuming and inefficient. A model-based optimisation and real-time control strategy that includes model output uncertainty could help in accelerating the primary drying phase while controlling the risk of failure of the critical quality attributes (CQAs). In each iteration of the real-time control strategy, a design space is computed to select an optimal set of CPPs. The aim of the control strategy is to avoid product structure loss, which occurs when the sublimation interface temperature ($T_{i}$) exceeds the the collapse temperature ($T_{c}$) common during unexpected disturbances, while preventing the choked flow conditions leading to a loss of pressure control. The proposed methodology was experimentally verified when the chamber pressure and shelf fluid system were intentionally subjected to moderate process disturbances. Moreover, the end of the primary drying phase was predicted using both uncertainty analysis and a comparative pressure measurement technique. Both the prediction of $T_{i}$ and end of primary drying were in agreement with the experimental data. Hence, it was confirmed that the proposed real-time control strategy is capable of mitigating the effect of moderate disturbances during batch freeze-drying.

## 1. Introduction

Pharmaceutical freeze-drying or lyophilisation is a dehydration process mainly used for stabilizing parenteral therapeutical agents contained in aqueous solutions. By removing most of the water, the shelf-life of the product is prolonged significantly because water drives many destabilization pathways. Since, freeze-dying is a low-temperature process it is a very popular processing technique for heat-labile biological drug substances. Therefore, many of the approved biopharmaceuticals by the FDA and EMA are stabilized by lyophilisation [1]. However, freeze-drying is a long and costly process that require a lot of aseptic floor space [2].

The freeze-drying process consists of multiple consecutive phases. After loading the vials, the shelf temperature is lowered gradually to freeze the liquid in the vials. Next, the chamber pressure ($P_{c}$) is decreased (70 to 1 Pa) to establish the primary drying phase, enabling the sublimation of all the ice and formation of a porous network. However, there are some constrains in this phase. At first, the shelf temperature should be balanced well to supply heat for sublimation while not exceeding a critical product temperature ($T_{c}$). The $T_{c}$ is slightly higher then the glass transition temperature of the maximally freeze-concentrated amorphous solutes ($T_{g^{\prime }}$) or the eutectic temperature ($T_{e}$) in case of a crystalline system and can be determined with a freeze-dry microscope. Surpassing the $T_{c}$ would induce a loss of structure in the dried layer [3,4]. The second constraint is the choked flow condition (${\dot{m}}_{sub,chok}$). When the sublimation rate exceeds this limit it will result in a loss of pressure control of the freeze-dryer. During this phenomena, the vapour flow is to high for the dimensions of the limiting channels (vial neck or condenser duct) of the freeze-dryer system resulting in a compression of the gas and a undesirable pressure build up at the ice sublimation interface [4,5]. The loss of pressure control also means a loss of control over the heat transfer, which could in turn lead to collapse when surpassing the $T_{c}$. The last freeze-drying phase is secondary drying. It is initiated when all ice is removed by increasing the shelf temperature ($T_{s}$) to start desorption of residual bounded water [6,7].

Nowadays, the typical way of operating a batch freeze-dryer is based on fixed manufacturing protocols. This means that all freeze-drying phases are programmed sequentially based on predefined timings while maintaining, or at best linearly varying, the critical process parameters (CPPs) (i.e., $P_{c}$ and $T_{s}$) in between defined setpoints of the protocol. The reason being that many of the industrially applied freeze-drying protocols were originally determined based on trial-and-error rather than mechanistic knowledge. This also makes that the occurrence of unexpected disturbances during processing are not compensated for by adjusting the CPPs. Failure of the freeze-drying process, sometimes resulting in a costly total loss of the batch, is therefore not uncommon. Indeed, for commercial manufacturing all vials are still visually checked for failures while only a representative sample is sent for offline quality control to evaluate its critical quality attributes (CQAs). Hence, there exists ample room for improvement of the economic performance of most freeze-drying operations.

Supervisory control and optimisation of the freeze-drying process could avert product failures from happening. Special attention should go to the primary drying phase as it is typically the longest phase of the freeze-drying process. Hence, optimization of primary drying can significantly reduce the total processing time and thus also operating costs while improving product quality. A solution exists in dynamically adjusting the CPPs of the primary drying process, i.e., chamber pressure ($P_{c}$) and shelf temperature ($T_{s}$), in order to compensate for process disturbances as well as the continuously changing physical state of the product, i.e., increasing dried layer thickness [4,8].

However, in order to establish such supervisory strategies, adequate measurements are required in real time. In the case of freeze-drying, the temperature at the sublimation front ($T_{i}$) is the most critical parameter as it determines the sublimation rate and should not surpass the critical temperature. Because the sublimation front moves from the top to the bottom of the product during primary drying, it is not possible to measure this sublimation front with a fixed temperature probe. Moreover, the presence of probes will alter the thermodynamics of the measured vial, might damage the product and is not representative for the entire batch. An alternative is to use mathematical models describing the physical mechanisms of heat and mass-transfer occurring [9,10,11,12,13]. To obtain information on $T_{i}$, such mechanistic models can then be applied as a soft sensor if the models are fed with real-time information of the system (i.e., $P_{c}$, $T_{s}$, dried layer thickness ($L_{dried}$)). Moreover, models make it possible to drive the process continuously towards its performance boundaries with the ability to control the risks of failures [14].

Mechanistic models have intrinsic model output uncertainty due to model simplifications and assumptions. In addition to that, there also exists natural variation of certain model parameters causing additional uncertainties [15]. It is the novelty of this proposed paper to carefully characterize all these variabilities and to implement them real-time using a model-based optimisation and control strategy, to yield robust model predictions. By continuously performing model output uncertainty analysis, taking into account different sources of uncertainty, a dynamic design space is constructed during the primary drying phase of a lab-scale freeze-dryer. This design space makes it possible to optimise the primary drying process in real time while taking into account process constraints. Hereto both the shelf temperature and the chamber pressure are automatically adjusted. Moreover, by including systematic checks on the manipulated variables, the proposed strategy ensures that the process is under control even in the case of unexpected disturbances.

## 2. Materials and Methods

### 2.1. Supervisory Control of the Freeze-Dryer

The optimisation and control strategy was developed on an Amsco-Finn Aqua GT4 freeze-dryer (GEA, Köln, Germany) which was retrofitted with PR-4114 programmable logic controllers (PLC) (PR electronics, Rønde, Denmark) and a Pro-Face AGP3000 (Schneider Electric, Rueil-Malmaison, France) Human-Machine Interface with Modbus TCP/IP communication capabilities. To keep the condenser temperature, shelf temperature and chamber pressure at their predetermined setpoints, multiple ON-OFF process control loops were programmed on the PLC. Hereto the freeze-dryer is supplemented with resistance temperature detectors in the condenser and shelf fluid system, and two different pressure gauges in the drying chamber. To measure the product temperature directly three additional thin gauge type-K thermocouples (Conrad Electronic, Hirschau, Germany) were available in the drying chamber. To measure chamber pressure both a Pirani and Capacitance pressure gauge are present, yielding the opportunity to apply a comparative pressure measurement methodology [16]. Due to the difference of the measurement principle of the two pressure gauges, it is possible to monitor the gas composition in the freeze-drying chamber. If the gas composition shifts from a predominantly water vapour environment, i.e., during sublimation, to an environment mainly composed of nitrogen gas, i.e., at the end of primary drying, then the pressure signal of the Pirani gauge will approximate the value obtained with the Capacitance sensor [17]. Note however that the chamber pressure ($P_{c}$) is always controlled by the signal coming from the capacitance pressure since it is the most accurate sensor. Unless mentioned otherwise, the machine operating limits of the freeze-dryer were set to −40 ${}^{\circ }$C and 50 ${}^{\circ }$C for the shelf fluid system with a maximum cooling rate of 0.8 ${}^{\circ }$C/min and maximum heating rate of 1 ${}^{\circ }$C/min. The ON-OFF temperature controller of the cooling fluid was set to have a deadband of 1.5 ${}^{\circ }$C. The vacuum pump could operate down to 7 Pa but for this study the lower limit was set at 10 Pa with a deadband of 0.4 Pa.

For the the purpose of supervisory control and data acquisition (SCADA), an additional remote computer was installed with LabVIEW 2017 including the NI-DSC module (National Instruments, Austin, USA) to communicate with the freeze-dryer PLC using Modbus TCP/IP. A state-machine based application was developed in the LabVIEW software to drive the freeze-dryer through all sequential freeze-drying steps, i.e., freezing, condenser preparation, primary drying initialization, primary drying with optimisation and control, secondary drying and venting/stoppering. Please note that the proposed strategy used for primary drying was implemented in MATLAB 2018a using the Parallel Computing Toolbox (Mathworks, Natick, USA) which had to be integrated in the LabVIEW application.

### 2.2. Primary Drying Model

A mechanistic model describing the primary drying phase of pharmaceutical vials in a traditional batch freeze-dryer is the core of the real-time optimisation and control strategy. This mechanistic model has been described thoroughly in the literature [8]. The model consists of a system of equations which have to be solved simultaneously (Equations (1)–(3)). It describes the gradual increase from the top to the vial bottom of a planar dry layer on top of the frozen product. It is based on a mass and heat balance assuming that all energy is used for the sublimation process, hence presuming steady state conditions. The model is characterised by five parameters and three variables (Table 1). As mentioned before, each of these contributes in their own way to the model output uncertainty. The model outputs of interest in this study are the sublimation interface temperature $T_{i}$ [K], the dry layer thickness $L_{dried}$ [m] and the sublimation rate ${\dot{m}}_{sub}$ [kg/s].

(1) $$e^{A_{P_{i}}-\frac{B_{P_{i}}}{T_{i}}+C_{P_{i}}ln\left(T_{i}\right)-D_{P_{i}}T_{i}}=-\frac{-A_{p}\Delta H_{sub}P_{c}-A_{v}K_{v}R_{p}MT_{s}+A_{v}K_{v}R_{p}MT_{i}+A_{v}K_{v}R_{p}M\Delta T}{A_{p}\Delta H_{sub}}$$

(2) $$\Delta T=\frac{a\frac{\left(L_{tot}-L_{dried}\right)\left(P_{i}-P_{c}\right)}{R_{p}}-b\left(L_{tot}-L_{dried}\right)\left(T_{s}-T_{i}\right)}{1-b(L_{tot}-L_{dried})}$$

(3) $$\Delta H_{sub}=A_{H_{sub}}+B_{H_{sub}}T_{i}-B_{H_{sub}}T_{i}^{2}+C_{H_{sub}}e^{-{\left(\frac{Ti}{D_{H_{sub}}}\right)}^{2}}$$

Here, $A_{P_{i}}$, $B_{P_{i}}$, $C_{P_{i}}$ and $D_{P_{i}}$ are coefficients to describe the relationship between ice temperature and its partial vapour pressure ($P_{i}$), *a* and *b* are constants to convert to SI-units [18], $A_{p}$ and $A_{v}$ are respectively the product and vial surface area [m${}^{2}$], $\Delta H_{sub}$ is the latent heat of ice sublimation [J/mol], $K_{v}$ the heat transfer coefficient [J/m${}^{2}$s·K], $R_{p}$ the dry layer resistance [m/s], *M* the molecular weight of water [kg/mol], ΔT the temperature difference across the ice layer [K], $L_{tot}$ the total thickness of the dry layer [m], $L_{dried}$ the thickness of the dry layer [m] and coefficients $A_{H_{sub}}$, $B_{H_{sub}}$, $C_{H_{sub}}$, $D_{H_{sub}}$ and $E_{H_{sub}}$ describe the empirical relationship between $\Delta H_{sub}$ and $T_{i}$ [19].

Furthermore, the maximum allowable sublimation rate ${\dot{m}}_{sub,chok}$ [kg/s] was calculated by applying a chocked flow model [20]. The latter is based on the vial neck diameter $r_{n}$ and the smallest diameter of the butterfly valve on the duct between the condenser and drying chamber $r_{d}$.

### 2.3. Input Parameters and Variability Estimation

The vial inner radius $r_{i}$ and outer radius $r_{o}$ as well as the filling volume $V_{fill}$ are parameters of the mechanistic primary drying model that are intrinsically distributed. In this case, the parameter values and their variability were determined at the start of the process and fixed during the entire progression of the primary drying phase. The heat transfer coefficient $K_{v}$ and the dry layer resistance $R_{p}$ were next to their intrinsic variability also dependent on the variability of other model parameters since they are estimated from respectively $P_{c}$ and $L_{dried}$. The other three sources of uncertainty and their variability ($T_{s}$, $P_{c}$ and $L_{dried}$) were updated regularly during optimisation and control.

#### 2.3.1. Determination of the Heat Transfer Coefficient

The heat transfer coefficient $K_{v}$ describes the efficiency of the heat transfer from the technical fluid inside the shelves to the ice in the vial and is therefore dependent on the freeze-dryer design and vial type. In this work, $K_{v}$ was determined using a gravimetric method at five different pressure levels. To this end, forty nine 10R vials (Schott AG, Mainz, Germany) filled with 3 mL of deionised water were weighed and stacked in a 7 × 7 hexagonal pattern and placed on the middle shelf of the freeze dryer. The product temperature was monitored using four thin gauge type-K thermocouple (Conrad Electronic, Hirschau, Germany) which were randomly divided between the edge and centre vial population. Next, the vials were equilibrated at 3 ${}^{\circ }$C and frozen to −30 ${}^{\circ }$C with a cooling rate of approximately 1 ${}^{\circ }$C/min which was maintained for 2 h. Then, a vacuum was pulled to the desired setpoints (7, 10, 15, 20, and 25 Pa) after which the $T_{s}$ was gradually increased to −20 ${}^{\circ }$C during a period of 10 min. After 6 h of primary drying, the sublimation process was abruptly interrupted by venting the vacuum chamber. All vials were stoppered immediately. Afterwards, the vials were reweighed in order to calculate the $K_{v}$ of every single vial according to Equation (4).

(4) $$K_{v}=\frac{m_{sub}\Delta H_{sub}}{A_{v}M\int_{t_{0}}^{t_{end}}\left(T_{s}-T_{b}\right)dt}$$

With $m_{sub}$ the sublimated mass [kg], $t_{0}$ and $t_{end}$ respectively the start and end time of primary drying [s] and $T_{b}$ the product temperature at the bottom of the vial [K]. Please note that there exists a significant increase in radiative power towards vials at the edge of the stack as they are not completely shielded from the door and walls of the freeze-dryer. This is why the vials were split in an edge group, i.e., all vials who do not have six direct neighbours, and a centre group, i.e., vials who were in direct contact with six neighbouring vials, for the $K_{v}$ determination. The mean $K_{v}$ as well as the relative standard deviation (RSD) were calculated for each group at the five different pressure levels. By using Equation (5), $K_{v}$ could be described in function of the chamber pressure using the α [J/m${}^{2}$s·K], β [J/m${}^{2}$s·KPa] and γ [1/Pa] parameters. These parameters were determined for both groups of vials using a weighted nonlinear regression, with the inverse of the RSD as the weights. At last, the pooled RSD over all pressure levels was computed as a degree of variation for the $K_{v}$ parameter.

(5) $$K_{v}=\alpha +\frac{\beta P_{c}}{1+\gamma P_{c}}$$

#### 2.3.2. Determination of the Dry Layer Resistance

The dry layer resistance $R_{p}$ [m/s] is a measure of the vapor flow impedance as a result of the dried layer micro-structure. Since the dried layer thickness $L_{dried}$ [m] increases during primary drying, $R_{p}$ is also changing over time. This relationship is expressed empirically in Equation (6) with $R_{p0}$ [m/s], $A_{Rp}$ [1/s] and $B_{Rp}$ [1/m] coefficients that require calibration using experimental data.

(6) $$R_{p}=R_{p0}+\frac{A_{Rp}L_{dried}}{1+B_{Rp}L_{dried}}$$

To determine these coefficients as well as the variability of $R_{p}$, two identical freeze-drying runs were performed. During these experiments three centre vials were monitored with a thin gauge thermocouple at the bottom-centre of the vial. A stack of vials was loaded and treated as described in Section 2.3.1 but with 3 mL of a 3% m/V sucrose solution (Fagron, Nazareth, Belgium). The pressure was set to 10 Pa for the full length of the primary drying phase. By applying Equation (7) throughout (13), the $R_{p}$ and $L_{dried}$ profiles could be calculated. Please note that the partial vapour pressure of ice $P_{i}$ [Pa] is expressed as a function of the sublimation front temperature $T_{i}$ [K] by the empirical Equation (9). Next, $T_{i}$ was estimated from the product temperature at the bottom of the vial $T_{b}$ using a heat conduction model (Equation (10)). Finally, the progress of $L_{dried}$ was calculated from the $m_{sub}$ (Equation (13)).

(7) $$R_{p}=\frac{\left(P_{i}-P_{c}\right)A_{p}}{{\dot{m}}_{sub}}$$

With $A_{p}$ the product surface area [m${}^{2}$].

(8) $${\dot{m}}_{sub}=\frac{K_{v}\left(T_{s}-T_{b}\right)M}{\Delta H_{sub}}$$

(9) $$P_{i}=e^{A_{P_{i}}-\frac{B_{P_{i}}}{T_{i}}+C_{P_{i}}\ln\left(T_{i}\right)-D_{P_{i}}T_{i}}$$

(10) $$T_{i}=T_{b}-\frac{\left(T_{s}-T_{b}\right)K_{v}L_{ice}}{\lambda_{ice}}$$

With $\lambda_{ice}$ the thermal conductivity of ice [W/mK].

(11) $$L_{ice}=L_{tot}-L_{dried}$$

(12) $$L_{tot}=\frac{V_{fill}\rho_{sol}\epsilon }{A_{p}\rho_{ice}}$$

(13) $$L_{dried}=\frac{m_{sub}}{\rho_{ice}\epsilon A_{p}}$$

With $V_{fill}$ the filling volume [m${}^{3}$], $\rho_{sol}$ the density of the solution [kg/m${}^{3}$], ϵ the porosity of the dried layer [-] and $\rho_{ice}$ the density of ice [kg/m${}^{3}$].

Once the six $R_{p}$ and $L_{dried}$ curves were obtained, a non-linear regression was performed to calibrate the $R_{p0}$, $A_{Rp}$ and $B_{Rp}$ parameters of Equation (6). Please note that negative $R_{p}$ and temperature values at the end of primary drying, i.e., where the thermocouple value is shifted upwards due to loss of contact with ice, were omitted from the data set as these points are not representative. Afterwards, all the $R_{p}$ data were segmented in 25 equidistant $L_{dried}$ bins and a standard deviation was calculated for each of the bins. All 25 $R_{p}$ standard deviations were finally pooled to get a degree of variation for the dried layer resistance parameter.

#### 2.3.3. Determination of Filling Volume Variability

The variability of the vial filling volume $V_{fill}$ [m${}^{3}$] was measured by determining the tare weight of 100 vials, sequentially filling them with 3 ml of deionised water using a Handystep pipettor (Brandtech, Essex, CT, USA) and reweighing them. Next the $V_{fill}$ per vial was calculated by dividing the weight of the water with the density of water at 25 ${}^{\circ }$C (i.e., 997 kg/m${}^{3}$). Lastly, the standard deviation of those volumes was calculated to yield a number for the $V_{fill}$ variation.

#### 2.3.4. Determination of Vial Radii Variability

The variability of the outer radius $r_{o}$ [m] of a vial was estimated by measuring the outer diameter of 100 10R Schott vials with a 10 μm resolution caliper and subsequently calculating the standard deviation. Because the vial neck hampered an accurate measurement of the inner diameter, the same degree of variability was assumed for the inner radius $r_{i}$ [m].

#### 2.3.5. Determination of the Critical Temperature

The critical temperature $T_{c}$ [K] is the maximum allowable temperature of the sublimation front. It should be noted that this characteristic temperature is highly dependent on the product formulation. When the temperature of the dry layer exceeds $T_{c}$ with only a minor fraction for a certain period of time, micro-collapses can occur which will change the micro-structure of the dry layer as well as its vapor flow resistance. In the case of extensive product temperature excursions during primary drying, macro-collapse or eutectic melt can occur which will impair the quality of the product. In order to determine this critical threshold, $T_{c}$ was investigated using a freeze-dry microscope (Linkam Scientific, Tadworth, UK). In this case 5 μL of the formulation used in this study was loaded on the system and the sample was frozen down to −40 ${}^{\circ }$C for 5 min. Next, a vacuum of 1 Pa was introduced and the sample was equilibrated at −36 ${}^{\circ }$C for another 4 min followed by small step increases of 0.5 ${}^{\circ }$C up to −32.5 ${}^{\circ }$C. Each step was attained for two minutes. At the end an image of the sample was taken to determine the exact location of the sublimation front. Based on the image, $T_{c}$ was determined as the highest temperature without any significant structural changes in the micro-structure of the dried layer.

#### 2.3.6. Determination of the Pressure Decrease Curve

Since sublimation requires a deep-vacuum, the chamber pressure should be lowered at the start of the primary drying phase using a vacuum pump. The initial pressure drop from atmospheric pressure until the start of ice sublimation was predefined taking the machine limitations into account. To have a robust estimation of this characteristic pressure decrease curve, ten repetitive runs were performed between atmospheric pressure and the minimal achievable pressure (7 Pa). Above 130 Pa, the Pirani sensor value $P_{c,Pir}$ was used instead of the signal from the capacitance sensor $P_{c,Cap}$ as this is the maximum limit for the latter. The slowest cumulative curve was chosen as the machine limit.

### 2.4. Freezing and Primary Drying Initialization Phase

The supervisory application programmed in LabVIEW starts with a freezing phase which is programmed according to a fixed protocol, i.e., list of setpoints for the shelf temperature $T_{s}$ at fixed time points need to be defined. The low-level temperature controller will subsequently aim for a linear transition between those setpoint values. At the same time, the condenser is activated and cooled to −50 ${}^{\circ }$C. An additional check was implemented at the last temperature setpoint in order to verify whether the desired $T_{s}$ was achieved and if the condenser temperature was below −40 ${}^{\circ }$C. If both conditions are satisfied, the application automatically continues to the initialization of the primary drying phase.

To start the primary drying initialization phase, the last $T_{s}$ setpoint from the preceded freezing phase was maintained and $P_{c}$ was decreased according to the preset pressure decrease curve (Section 2.3.6) with a time resolution of 10 s. At each time point the primary drying model was evaluated and checked for a positive ${\dot{m}}_{sub}$, i.e., to inquire if ice sublimation started. From the moment this was the case, the initialization phase was terminated and the model-based control strategy of the primary drying phase was initiated. However, in the case that no sublimation was perceived, $T_{i}$ was assumed to be equal to $T_{s}$ while $L_{dried}$ and ${\dot{m}}_{sub}$ were maintained at zero.

### 2.5. Primary Drying

#### 2.5.1. Uncertainty Analysis

Through the application of uncertainty analysis (UA) to the primary drying model (Section 2.2), a design space was obtained for the primary drying phase at fixed intervals in time. Hence, the term dynamic design space. The methodology used for the UA is based on the work of Mortier et al. with some important changes [8]. These modifications were introduced to decrease the computational load of the proposed model-based strategy. This makes it possible to perform all calculations in real time on a desktop computer with a state-of-the-art processor.

The UA starts off by defining a machine capability space. This space spans the multivariable combination of the manipulated variables which can be achieved taking into account the previous operating setpoint. Please note that in this case the two manipulated variables of interest are $P_{c}$ and $T_{s}$. Obviously, the size of this space depends on the limits of the shelf fluid system, the vacuum pump and the considered temporal resolution expressed as Δt. This machine capability space is subsequently transformed into a grid, which considers only a limited number of combinations in $P_{c}$ and $T_{s}$. To this end, a resolution of roughly 0.2 Pa and 0.5 K was chosen.

Next, Monte Carlo simulations are performed to estimate the uncertainty of the primary drying model outputs. Accordingly, a model parameter space is constructed by sampling a fixed number of parameter combinations $n_{sample}$ within the predetermined variability of all model inputs (see Section 2.2). For $r_{o}$, $r_{i}$, $K_{v}$, $R_{p}$ and $V_{fill}$ a normally distributed random sampling method is applied using the (relative) standard deviation as a measure of variation. In contrast, the manipulated variables $P_{c}$ and $T_{s}$ were sampled uniformly within the machine capability space enlarged with their respective variations using a Sobol sampling technique. The samples for the $L_{dried}$ variable are obtained through error propagation. Meaning that the predicted distribution of $L_{dried}$ in the previous sampled time point is reconstructed trough a fit with the Pearson system using its first four moments, i.e., mean, variance, skewness and kurtosis, followed by a random sampling from the fitted distribution type. The Pearson system includes seven distribution types and categorizes a dataset using these moments [21]. Note that there is no covariance assumed between all eight input parameters. Hence, the samples were randomly combined to achieve an eight-dimensional model parameter space.

All points within this parameter space were subsequently used to solve the primary drying model for $T_{i}$ and $L_{dried}$. Moreover, $L_{tot}$ was calculated by employing Equation (12) which was later compared to the resultant $L_{dried}$ to check for completion of the primary drying phase ($L_{end}$ [-]). Hence, a three-dimensional model output space composed of $T_{i}$, $L_{dried}$ and $L_{end}$ is obtained as a solution to the eight-dimensional model parameter space.

**Figure 1:**
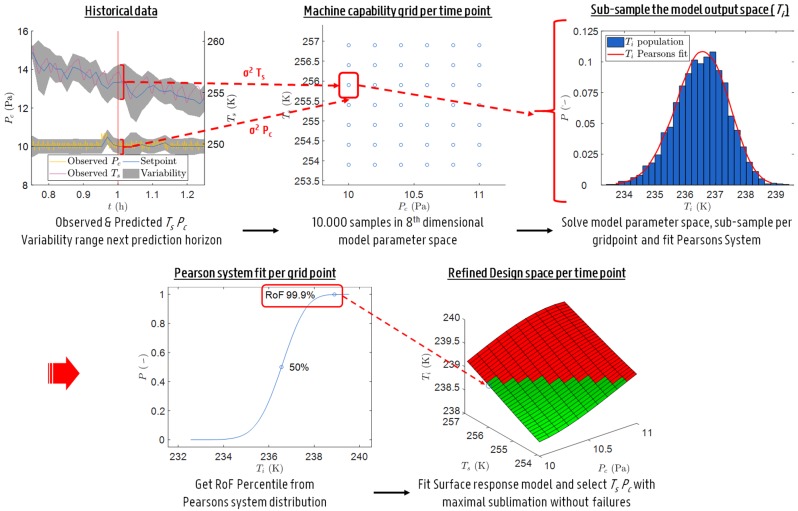
Schematic representation of the methodology used for the uncertainty analysis.

In the next step, the model output space was sub-sampled using the machine capability grid of $P_{c}$-$T_{s}$ combinations. This is achieved by selecting all solutions in the model output space that originates from $P_{c}$-$T_{s}$ combinations which lay in the enlarged area, defined by the $P_{c}$ and $T_{s}$ variation, around each machine capability grid point (cf. Figure 1). This resulted in a model output sub-population for each grid point in terms of $T_{i}$, $L_{dried}$ and $L_{end}$. Please note that $P_{c}$ and $T_{s}$ are distributed uniformly in the model parameter space. Hence, a sub-sample based on $P_{c}$ and $T_{s}$ of that model parameter space yields comparable distributions for the other six sources of variation in the model [22]. Which makes that a representative sub-population is taken from the model output space for each grid point.

For each grid point the sub-population in $L_{dried}$ was used to calculate the first four moments of its distribution to propagate the error on $L_{dried}$. Next, the percentage of $L_{end}$ was computed and evaluated whether the end of drying was reached. Moreover, a risk of failure (RoF) space was calculated based on the distributed $T_{i}$ solutions for each of the considered grid points of the machine capability space. To do this, a Pearson distribution fit is made of all $T_{i}$ sub-populations, using the first four moments, and the 1-RoF upper percentiles (i.e., uncertainty degree) of these fitted distributions are determined to yield that RoF space. The RoF space, with a coarse resolution of 0.2 Pa and 0.5 K, explains the $T_{i}$ associated with a user defined RoF for all $P_{c}$-$T_{s}$ combinations. In order to further refine these results, a cubic surface response model was fitted through this 3-dimensional data and re-evaluated with a finer resolution of 0.1 Pa and 0.1 K.

Ultimately, the refined grid was used to evaluate the primary drying model together with nominal input parameters to obtain an estimate of ${\dot{m}}_{sub}$. Later the failure modes were assessed. Those combinations with a ${\dot{m}}_{sub}$ higher or equal to ${\dot{m}}_{sub,chok}$, or a $T_{i}$ associated with a certain RoF above or equal to the $T_{c}$, are invalidated. Hence, a design space was constructed which was valid for that sampling instance.

#### 2.5.2. Optimisation and Control of the Primary Drying Phase

Based on the dynamic design space constructed by the UA it is possible to control and optimise the primary drying phase of a freeze-drying process taking into account a defined and accepted risk of failure (RoF). To do so, a supervisory control strategy is implemented on top of the existing low-level control loops of the freeze dryer, i.e., the ON-OFF controllers for $T_{s}$ and $P_{c}$. Because the proposed strategy makes direct use of model output uncertainty it can be referred to as a robust model-based strategy for the purpose of supervisory optimisation and control. In fact, the result of the UA on the primary drying model is used as the cost function which is optimised towards the maximization of the sublimation rate ${\dot{m}}_{sub}$ while not exceeding a predetermined RoF as inferred from $T_{i}$ and ${\dot{m}}_{sub}$ predictions.

The presented strategy is based on the receding horizon principle, i.e., optimal process conditions in the near future are calculated using information of the past and a cost function. Depending on the chosen sampling interval Δt the prediction horizon is divided in multiple prediction points. For each of these points in time a machine capability space is constructed wherein the UA was performed (Section 2.5.1). Please note that this machine capability space is dependent on the selection of the optimal $P_{c}$-$T_{s}$ combination in the previous prediction point. The result is a dynamic design space for the considered horizon. Next, the set of manipulated variables, i.e., the combination of $P_{c}$ and $T_{s}$, associated with the highest ${\dot{m}}_{sub}$ in this design space are selected as the optimal settings for the considered prediction points.

For each optimisation in the receding horizon, the variability of the manipulated variables $P_{c}$ and $T_{s}$ as well as the variability in $L_{dried}$ has to be included in the UA. The former are estimated by taking into account the absolute difference between the historically measured signals and their applied setpoints during the previous prediction horizon. Whereas variation on $L_{dried}$ is estimated using the error propagation methodology described in Section 2.5.1. It is important to note that the distribution of $L_{dried}$ is propagated to the next prediction point via its first four moments of that distribution combined with random sampling from the Pearson system. Moreover, the prediction of $T_{i}$ and $L_{dried}$ are corrected after each horizon using the actual executed $P_{c}$ and $T_{s}$ in the primary drying model. Finally, the contributions of the other five sources of variability in the primary drying model were all determined upfront (Section 2.3) and therefore these values are fixed for intermittent predictions.

Besides the optimisation of ${\dot{m}}_{sub}$ through the use of the dynamic design space, the proposed supervisory strategy is also capable of handling unexpected disturbances in its manipulated variables, i.e., $T_{s}$ and $P_{c}$. In practice it is possible that pressure and temperature excursions occur even though a control system is in place. For example when a vacuum leak is present, when the vacuum valve is temporally sticking, or when uncontrolled disturbances in the fluid system impede the shelf temperature controller. In order to prevent these, a system check is performed before constructing the machine capability space, i.e., it was checked if the low-level controllers of the manipulative variables achieved in attaining the setpoint at the start of each new prediction horizon. In the case of a disturbance larger than 1.5 ${}^{\circ }$C or 0.4 Pa, the respective variable is restricted in the machine capability space and therefore fixed to its current measured value. In such case, the process is only optimised in the direction of the non-restricted variable. Please note that if this restriction would lead to a design space without options within the considered RoF level, the most conservative setting of the machine capability space is automatically selected (i.e., the lowest $P_{c}$ or $T_{s}$). Consistently choosing this conservative option will obviously decrease ${\dot{m}}_{sub}$, and thus also $T_{i}$, and will therefore result in a valid design space over time. In this work, the lowest authorized pressure was chosen to be 8 Pa in case of a fluid system disturbance. If the disturbance were passed, normal operation conditions were resumed.

It is important to remember that two groups of vials were identified earlier based on their distinct heat transfer properties (Section 2.3.1). This explains why in a first stage the proposed supervisory strategy is only targeted at the edge vials as these have higher $K_{v}$ values resulting in a faster drying cycle associated with higher ${\dot{m}}_{sub}$ and $T_{i}$ values which should be controlled. Only when the whole edge vial population is dry, as determined by the uncertainty analysis, will the target of process optimisation be shifted towards the centre vial population.

### 2.6. Primary Drying Endpoint

Once the end of primary drying is reached, the optimisation and control strategy is automatically stopped. Please note that both the comparative pressure measurement and the uncertainty analysis have to indicate the end of primary drying before starting secondary drying. In this case the pressure signals from a Pirani gauge $P_{c,Pir}$ and a capacitance $P_{c,Cap}$ gauge are compared. The ratio of both is calculated $P_{ratio}$ [-] which is indicative to the molecular composition of the gas inside the vacuum chamber. If the $P_{ratio}$ is near 1.6 the chamber is predominantly filled with water vapour, i.e., primary drying is ongoing. In contrast, if the chamber is filled mainly with nitrogen gas, i.e., primary drying is completely finished, a $P_{ratio}$ near 1 is expected. Please note that the transition between the two extremes is a smooth trajectory that depends on the spatial homogeneity of ${\dot{m}}_{sub}$ across the freeze dried batch [20]. Please note that in this case the capacitance gauge is of superior quality as compared to the Pirani sensor, i.e., higher precision. As such, the end of primary drying, as defined by the comparative pressure measurement, is reached if the $P_{ratio}$ is below 1.07 for a period of minimum 15 min. Contrarily, the end of primary drying, as defined by the UA, is obtained when the percentage of vials having reached the $L_{end}$ condition is equal to the uncertainty degree (i.e., 1 − RoF). Moreover, the accuracy of the UA endpoint prediction is evaluated by comparing its prediction to the midpoint in the $P_{ratio}$ trajectory [17].

### 2.7. Secondary Drying Phase

The secondary drying phase is programmed according to a fixed protocol. $P_{c}$ is set to 10 Pa and $T_{s}$ is increased gradually from where the primary drying ended towards a new setpoint which is maintained until the end of secondary drying. At this point the vacuum chamber is partially vented with nitrogen gas and the vials are stoppered. Finally, the chamber is completely vented and the vials are unloaded.

### 2.8. Experimental Verification

To verify the proper functioning of the proposed optimisation and control strategy for primary drying, six independent freeze-drying runs were performed at a pre-defined accepted Risk of Failure level of 0.1%, i.e., the predictions for $T_{i}$ cross $T_{c}$ only 0.1% of the time. Each experimental run contains 49 vials of the 10R type. All vials were filled with 3 mL of a 3% m/V sucrose solution (Fagron, Nazareth, Belgium). The vial stack was loaded into the freeze-dryer with pre-cooled shelf at 3 ${}^{\circ }$C. Next, the freezing protocol was initiated by ramping down the temperature to −36 ${}^{\circ }$C at a rate of 1 ${}^{\circ }$C/min. Next, the vacuum pump and condenser were initiated while freezing was maintained for another 2 h. After applying the last freezing setpoint of the initialization phase, primary drying and optimisation were started. All parameter values used by the robust model-based control are listed in Table 2. Once the end of primary drying is detected, secondary drying is started by heating the shelf temperature up to 25 ${}^{\circ }$C at 0.25 ${}^{\circ }$C/min which is maintained for 4 h. At the end of each run, the vials are stoppered under an atmosphere of nitrogen gas. Each vial was visually checked for defects. In this case, a slight shrinkage of the cake, attributed to the amorphous properties of the pure sucrose formulation, is not considered a defect [23].

To verify sublimation front temperature predictions $T_{i}$ in real time, three thin gauge thermocouple-K probes were installed in each vial stack. Since the largest part of the primary drying process is controlled for edge group, it was chosen to mount two thermocouples in edge vials (row 4, vial 1 and row 7, vial 4). The third probe sampled a centre vial (row 4, vial 4) to verify the dynamics in the final part of the process which is directed towards the centre vial population. The thermocouples were placed at the bottom-centre of the vials from which $T_{i}$ can be calculated using Equation (10) [16]. During operation it was constantly verified if the measured $T_{i}$ was within the 99.9% uncertainty range as predicted by the UA.

Three out of six verification runs were performed without the application of intended process disturbances. This to solely demonstrate the functioning of the proposed real-time optimisation algorithm. For the other three runs, multiple disturbances were intentionally introduced both in the temperature and pressure loops of the freeze-dryers. The objective of these disturbed trials is to evaluate the performance of the system check introduced on the machine capability space. This should assure that the proposed real-time optimisation strategy is able to keep the product safe in the presence of uncontrolled events. Because these disturbances were introduced as disturbed setpoints to the low-level control loops, they are out of the direct control of the optimisation algorithm. These disturbances are therefore only noticed when $P_{c}$ or $T_{s}$ start diverging from the setpoints calculated by the algorithm. In the third verification run, two fluid problems are simulated. One at the start of primary drying, and one after 5 h of drying. The disturbances were introduced by keeping $T_{s}$ constant around respectively −17.5 ${}^{\circ }$C and −25.5 ${}^{\circ }$C for a complete hour. Later on, in between 5 and 19 h of the same run, multiple pressure system disruptions were emulated. This was done by varying the setpoint of the local controller $P_{c}$ in multiple steps between 10.9 and 15 Pa. In the final part of the primary drying process, another $T_{s}$ disturbance of 1 h was introduced by keeping $T_{s}$ constant to a setpoint of −21 ${}^{\circ }$C after 20.7 h of drying. For the other two verification runs, comparable disturbances were implemented.

## 3. Results

### 3.1. Input Parameters and Variability

#### 3.1.1. Determination of the Heat Transfer Coefficient

**Figure 2:**
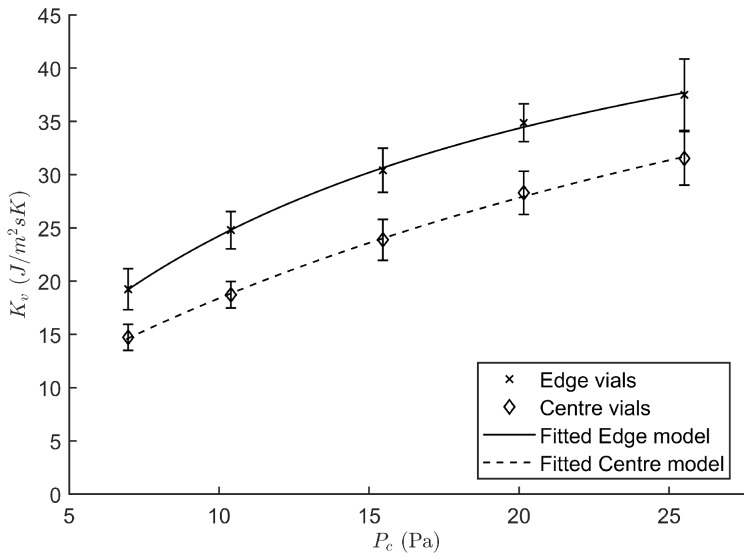
Heat transfer coefficient $K_{v}$ regressed against the chamber pressure $P_{c}$ for both the edge (cross) and centre vial (diamond) population.

The characteristic heat transfer coefficient $K_{v}$ was determined in function of the chamber pressure $P_{c}$ for both the edge and centre vials. Figure 2 shows the positive relation between $P_{c}$ and $K_{v}$, which is consistent with literature findings [8,20,24]. The reason being that a higher molecular density, at elevated chamber pressures, is associated with a higher convective heat transfer component of $K_{v}$. Please note that the vials have only limited contact with the shelf for conductive heat transfer due to their dome shaped vial bottom. Hence, the presence of a mediator gas between the vial and shelf surface can significantly contribute to additional convective heat transfer. Moreover, a consistently higher $K_{v}$ was found for the edge group as compared to the centre vials. This is because the edge vials are not completely shielded by the presence of surrounding vials, i.e., the edge vials have more field of view with the uncooled door and walls of the freeze-drying apparatus. This temperature difference drives a radiative heat flux which is consequently higher for the edge vial group [25]. This also explains why it was chosen to describe both vial groups with different regression equations (Equation (5)). For the edge group the nonlinear regression yielded a value of −1.14 J/m${}^{2}$s·K for $\alpha_{e}$ with a standard error of the estimate (SE) of 3.90, a $\beta_{e}$ of 4.46 J/m${}^{2}$s·KPa with a SE of 1.22 and a $\gamma_{e}$ of 0.0757 1/Pa with a SE of 0.0207. The centre group was described using an $\alpha_{c}$ of 3.46 J/m${}^{2}$s·K with a SE of 1.65, a $\beta_{c}$ of 1.93 J/m${}^{2}$s·KPa with a SE of 1.22 and a $\gamma_{c}$ of 0.0292 1/Pa with a SE of 0.0088. The RSD for both groups were comparable. Hence, only a single term of variation for the $K_{v}$ parameter was considered, i.e., $RSD_{K_{V}}$. Its value is calculated as the pooled RSD of all experiments and equals 0.0761. Due to their higher value of $K_{v}$, edge vials are more prone to collapse during the freeze-drying process. The focus of the proposed real-time optimisation strategy of the primary drying phase was therefore targeted mostly at the edge vials. Only when all edge vials are dried according to the model, was the $K_{v}$ parameter characteristic to the centre vials used for optimisation.

#### 3.1.2. Determination of the Dry Layer Resistance

The regression for $R_{p}$ against $L_{dried}$ was performed by applying Equations (7)–(13) on six different product temperature profiles. The resulting $R_{p}$ profiles were grouped and used for non-linear regression according to Equation (6). This leads to estimates of the regression parameters: $R_{p0}=1.51\;\times 10^{4}$ m/s (95 % CI: $1.27-1.75\;\times 10^{4}$), $A_{R_{p}}=9.68\;\times 10^{7}$ 1/s (95% CI: $8.78-10.58\;\times 10^{7}$) and $B_{R_{p}}=968$ 1/m (95% CI: 887−1049). Please note that the obtained $R_{p}$ profiles are very similar as compared to those found in the literature [26,27]. The profile shows a sharp increase in $R_{p}$ at the start of the primary drying process which tapers off near the end to a value of around $10\;\times 10^{4}$ m/s, i.e., the final resistance originating from the pores created by the ice crystals after sublimation. Please note that the water vapour inside the pores is assumed to be under Knudsen conditions meaning that the shape and dimensions of the pores had an influence on the evacuation of that water vapour out of the dried layer. The longer and narrower a pore is, the higher its resistance to water vapour.

**Figure 3:**
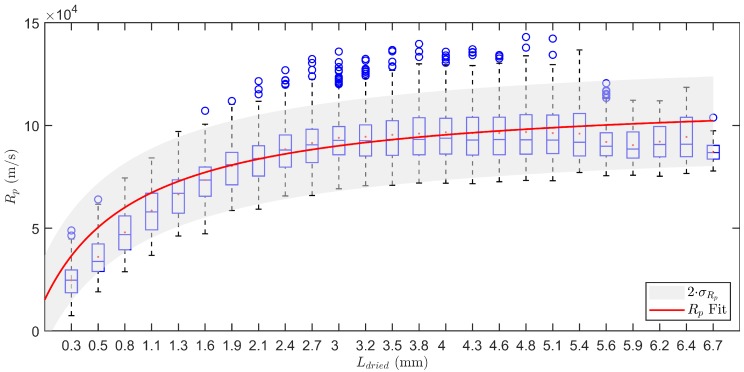
Boxplot representation of the dry layer resistance $R_{p}$ in function of the dry layer thickness $L_{dried}$. Data obtained during six individual measurements. The box represents the 25th up to the 75th percentile. The whiskers extend to the most extreme data points. Outliers are shown individually by the blue circles. Fit of the $R_{p}$ regression in red with an uncertainty band of 2 σ in grey.

The standard deviation of $R_{p}$ in 25 equidistant $L_{dried}$ bins was calculated and pooled. This results in $\sigma_{R_{p}}=1.10\;\times 10^{4}$ m/s. In Figure 3, the $R_{p}$ data is represented using a boxplot for each $L_{dried}$ bin. The fit of the regression curve enlarged by an interval of 2 times $\sigma_{R_{p}}$ is also shown. Please note that within the determined boundaries of variation the data is accurately described. Only for the first bins of $L_{dried}$ there is a slight overprediction.

#### 3.1.3. Variation of Vial Radii and Filling Volume

Variation on other model parameters (Table 1) was measured experimentally and described using the associated standard deviation. Please note that the distribution of the parameters were assumed to be normal. The standard deviation of the outer vial radius ($\sigma_{r_{o}}$) was measured with an caliper and equalled $5.44\;\times 10^{-5}$ m. For the inner vial radius ($\sigma_{r_{i}}$) a similar number was assumed. The filling volume variability was checked gravimetrically using deionised water. The standard deviation ($\sigma_{V_{fill}}$) equalled $1.29\;\times 10^{-8}$ m${}^{3}$.

#### 3.1.4. Determination of the Critical Temperature

**Figure 4:**
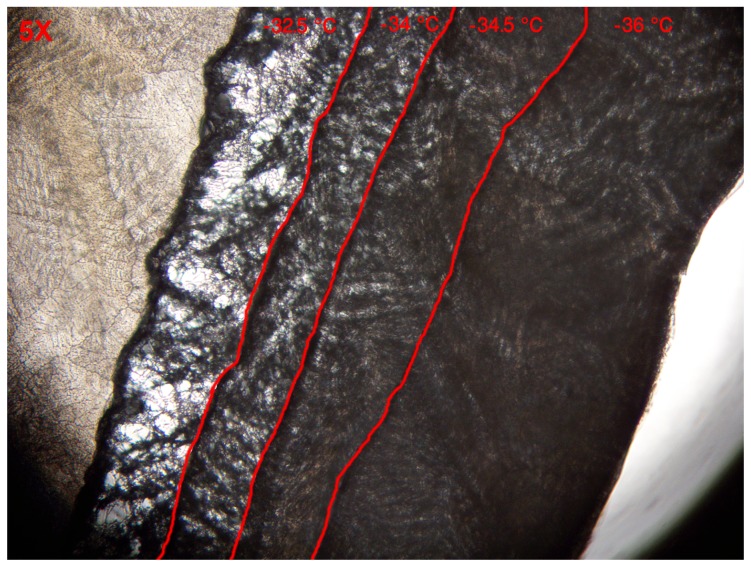
Overlay of images from the freeze-dry microscope at different temperatures. The red lines depicts the different temperature stages. Sublimation front moves from the right to the left side of the image.

$T_{c}$ was determined with the use of a freeze-drying microscope. Figure 4 depicts an overlay of images taken at then end of the temperature stage. The sublimation front moves from the right hand side towards the left and is marked in red in each overlay. Please note that ice crystals are still present on the left hand side of the image whereas the dark grey represents the dried material. From −36.0 to −34.5 ${}^{\circ }$C no abnormal changes in micro-structure were perceived. The shape of the pores in the dried layer followed the configuration of the ice crystals. However, when a temperature of −34 ${}^{\circ }$C was achieved a minimal change in pore shape and size was noticeable. The pore enlarged and material started to aggregate slightly. Pore enlargement is typically attributed to micro-collapse [28]. The dried product was near its $T_{g^{\prime }}$, allowing some degree of mobility however the total structure was not completely lost yet [29]. Upon heating the sample towards −32.5 ${}^{\circ }$C, collapse occurred. Because such micro-collapses are unwanted, as they alter the micro-structure of the pores and therefore also the $R_{p}$ coefficients, $T_{c}$ was defined to 238.9 K or −34.25 ${}^{\circ }$C.

### 3.2. Experimental Verification of the Real-Time Optimisation Strategy

#### 3.2.1. Verification Runs without Process Disturbances

Three normal operation verification experiments were performed to verify all functions of the primary drying optimisation and control strategy. The UA, using parallel computing, with 10,000 samples and a Δt of 60 s needs around 90 s to calculate a horizon of 120 s. Sub-sampling, fitting of a distribution to the $T_{i}$ sub-population and approximating the design space with a surface response model proved successful in reducing the computational load with a factor 40 as compared to the initial strategy proposed by Van Bockstal et al. [20].

**Figure 5:**
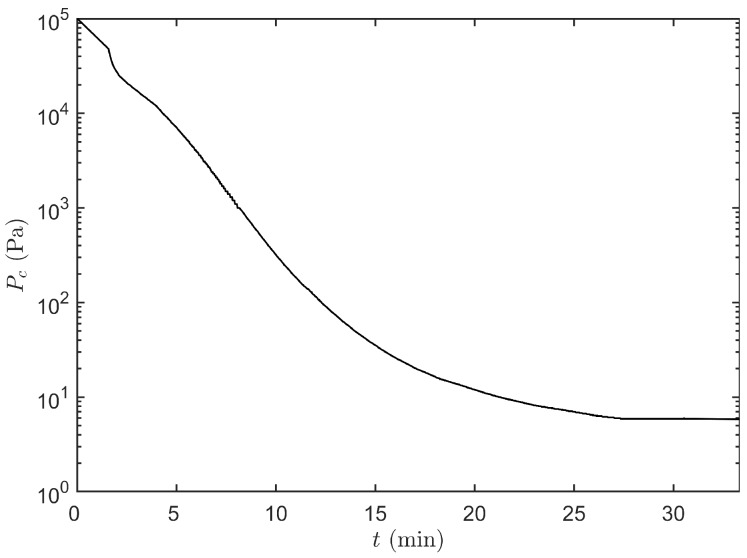
Trajectory of the chamber pressure $P_{c}$ in function of time *t* which is followed during the primary drying initialization phase.

**Figure 6:**
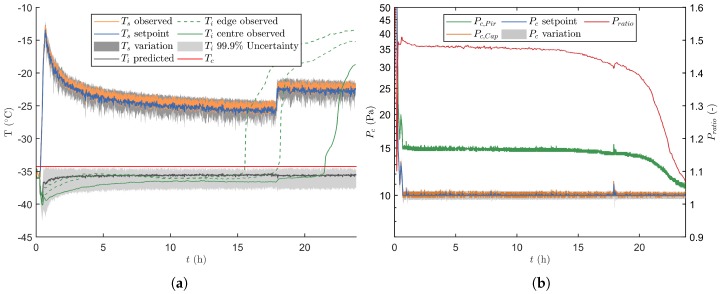
Second verification run without intended process disturbances. (**a**) Depiction of $T_{s}$ together with its associated variation, and $T_{i}$. The latter variable was either estimated by the primary drying model, or experimental measured for both the edge and centre vials. (**b**) The different $P_{c}$ signals are plotted in time together with its regularly updated variation.

After freezing and preparation of the condenser and vacuum pump, primary drying was initiated by lowering the pressure according to the pressure curve depicted in Figure 5. During this period $T_{s}$ was kept constant at −36 ${}^{\circ }$C. Whenever the primary drying model calculated a positive ${\dot{m}}_{sub}$, which is around 18 min at 14 Pa, the real-time optimisation strategy was initiated (Figure 6). At the start of primary drying a machine capability space was constructed around the initial operating point, i.e., $P_{c}=14$ Pa and $T_{s}=-36$ ${}^{\circ }$C. As these process settings are very conservative, the optimiser will first try to maximize ${\dot{m}}_{sub}$ by maximizing $T_{s}$ and by decreasing $P_{c}$. At around 27 min the strategy changed and $P_{c}$ was slightly increased, which boosts the convective heat flux and therefore also higher ${\dot{m}}_{sub}$. When 13.4 Pa was reached, at around 32 min, the calculated design space was partly limited by the constraint on $T_{i}$ and therefore the optimal $P_{c}/T_{s}$ combination was obtained by maximizing $T_{s}$ while slowly minimizing $P_{c}$ towards 10 Pa. Around 40 min the maximal heat flux was achieved which is limited by $R_{p}$. The highest ${\dot{m}}_{sub}$ with a 0.1% risk of failures, from that point forward was achieved by keeping $P_{c}$ at its minimal level while controlling the process through the adjustment of $T_{s}$. Because $R_{p}$ rises during the progression of primary drying, the value of $T_{s}$ was gradually decreased to maintain $T_{i}$ as close as possible to its upper limit as defined by $T_{c}$ as clearly shown in Figure 6.

**Figure 7:**
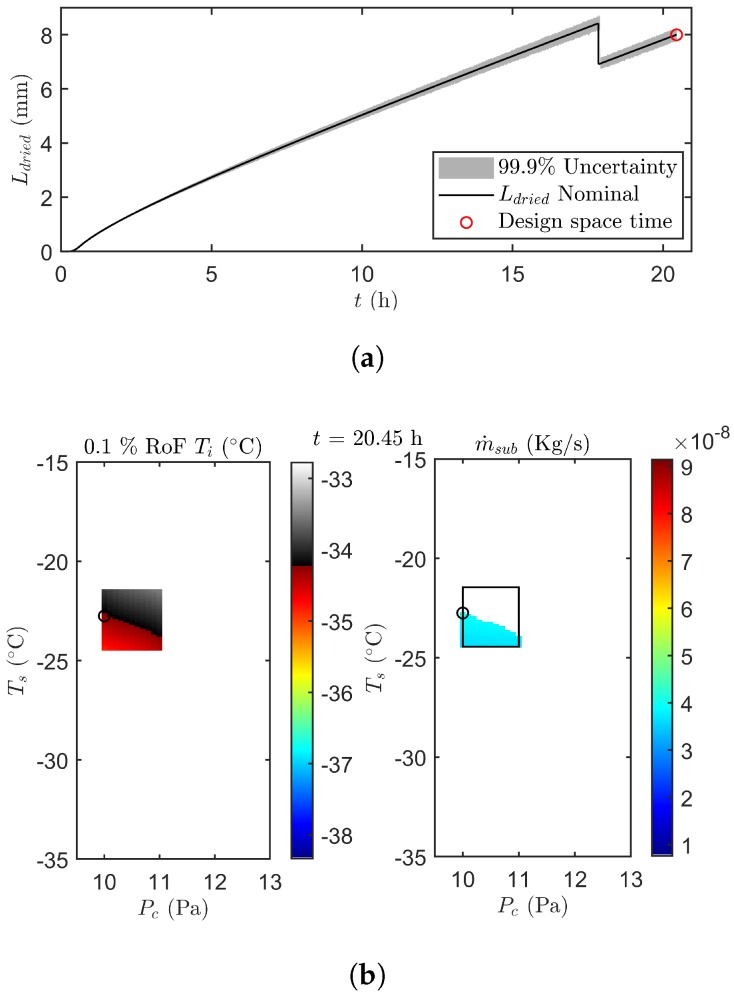
(**a**) The error propagation of $L_{dried}$ until time point 20.45 h (red circle). (**b**) Design space for a risk of failure of 0.1 % for $T_{i}$ (left) and ${\dot{m}}_{sub}$ (right) after 20.45 h of the second undisturbed verification run. Please note that the black box represents the machine capability space centered around the previous combination of manipulated variables. White and gray-scale points in the box are invalidated due to their high RoF. The black circle depicts the most optimal next operating point within the design space.

Figure 7 illustrates the results of the uncertainty analysis after 20.45 h of primary drying. The design space is created by invalidation of grid points with a $T_{i}$ at 0.1% RoF equal or above the $T_{c}$, and an ${\dot{m}}_{sub}$ above ${\dot{m}}_{sub,chok}$. Next, the point in the accepted design space with the highest ${\dot{m}}_{sub}$ is selected as the most optimal set of manipulative variables. The $L_{dried}$ population associated with that optimal grid point is subsequently propagated to the next prediction point as shown in Figure 7a. This results in a gradual increase of the uncertainty on $L_{dried}$ as primary drying progress.

Please note that after approximately 17.9 h in the primary drying phase, the UA estimated that all edge vials were dry. This because all the samples of the optimal grid point had a condition of $L_{end,edge}$ equal to 1. From that point onwards the $K_{v}$ coefficient of the centre vials was introduced in the calculations. Moreover the nominal $L_{dried}$ of the centre vials was used in the error propagation. Consequently, the predictions of $T_{i}$ and its associated uncertainty range was directed at the centre vials. This explains the sudden drop of $L_{dried}$ in Figure 7a. As the centre $K_{v}$ was also constantly lower as compared to the one obtained for the edge vials, a higher $T_{s}$ was employed to keep the heat flux at its upper limit. As a response $P_{c}$ was temporally increased to increase the heat flux even further, thereby reaching the new setpoint as fast as possible.

The absolute difference between the setpoints of the low-level ON-OFF controllers and the observed values were used as a term of variation for $T_{s}$ and $P_{c}$, which is indicated by the grey area around $P_{c}$ and $T_{s}$ in Figure 6. Please note that the measured value was always situated within this uncertain area and that it cycled around the setpoint value. This also indicates that all of the variability in the manipulated variables was fully covered by the uncertainty analysis.

By including the variation of eight different sources, the UA was capable of estimating an uncertainty on the calculated value of $T_{i}$. Please note that the nominal $T_{i}$ prediction is not centred in this uncertain area which indicates an asymmetric solution of the primary drying model. Moreover, the width of the predicted uncertain area decreases with the progress of drying as it is mainly linked to ${\dot{m}}_{sub}$. Indeed, ${\dot{m}}_{sub}$ decreases as primary drying is mainly limited by increasing $R_{p}$ values.

At last, an observed $T_{i}$ was also calculated. This value is obtained by correcting the measurements of the three thermocouples (Equation (10)). When focusing on the edge vials, which is the case from the start until 17.9 h, the observed $T_{i}$ is very close to the prediction of the nominal $T_{i}$ and is also inside the 99.9% uncertainty area. Similar observations can be made for the observed $T_{i}$ after this period which is aimed at the centre vials.

#### 3.2.2. Verification Runs with Process Disturbances

**Figure 8:**
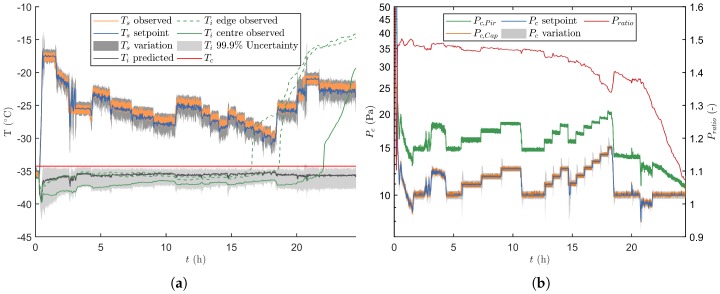
Third verification run with several intended $P_{c}$ and $T_{s}$ disturbances. (**a**) Depiction of $T_{s}$ together with its associated variation, and $T_{i}$. The latter variable was either estimated by the primary drying model, or experimental measured for both the edge and centre vials. (**b**) The different $P_{c}$ signals are plotted in time together with its regularly updated variation.

The results for a verification run with process disturbances is given in Figure 8. From these it is observed that the proposed control strategy correctly identifies the disturbance maximally two prediction horizons after its introduction, i.e., 240 s. At first, the variation of the disturbed manipulated variable increased since the absolute difference between its measurements and its setpoint value became bigger. This result is a broader $T_{i}$ distribution after the uncertainty analysis, which makes that a more conservative setting will be chosen to stay well below $T_{c}$. Furthermore, the disturbed dimension of the machine capability space is not taken into account when a disturbance occurs, i.e., its value is fixed to the measured value. This leads to a correction of the machine capability grid towards the disruption, which makes that the process is only controlled using the other non-disturbed manipulated variable. Such an event is for example observed after 0.5 h of primary drying. At this point ${\dot{m}}_{sub}$ is maximised by reducing $P_{c}$ from 13.2 to 8.8 Pa while maintaining the value of $T_{s}$ at −17.5 ${}^{\circ }$C because of the prior disturbances identified in this variable. Moreover, the variability on $T_{s}$ is increased at the onset of this event. Similar events can be noticed when $P_{c}$ is disturbed. For such an event the variability on $P_{c}$ is temporally enlarged while $T_{s}$ is lowered to keep the $T_{i}$ uncertainty under the critical limit. Notice that only with relatively severe process disturbances, for example occurring after 20.8 h, the 99.9% $T_{i}$ upper percentile briefly crossed the $T_{c}$ limit. However, this situation was quickly corrected, i.e., as fast as the freeze-dryer could operate by shifting the machine capability space to the most conservative grid point until a valid design space could again be achieved. Furthermore, the correction of $T_{i}$ and the $L_{dried}$ predictions in front of the UA kept future predictions on track.

Regarding the product temperature, a similar conclusions can be drawn for the disturbed verification runs as for the undisturbed runs. This begin the fact that for both the centre vials (i.e., from the start to 19.98 h of primary drying) and the edge vials (i.e., from 19.98 h until the end of primary drying), the observed $T_{i}$ could be kept inside its uncertainty area using the proposed real-time optimisation and control strategy.

#### 3.2.3. End of Primary Drying

In Figure 6 and Figure 8, the observed $T_{i}$ for the edge and centre vials started deviating before the predicted end of primary drying. This deviation is caused by an imbalance between the heat-transfer and ${\dot{m}}_{sub}$. Hence, at the end of primary drying the sublimation surface decreases as some parts of the vial are already dried. Consequently, the heat is not fully removed by the endothermic ice sublimation, leading to heating of the product [20]. Aside of the thermocouples, a comparative pressure measurement is also employed to determine the end of primary drying. This because near the end of sublimation the gas composition inside the drying chamber changes from water vapour towards nitrogen gas. Hence, the comparative signal or $P_{ratio}$ approaches a terminal value of 1. Both the midpoint and offset of the $P_{ratio}$ curve were compared to the time point at which 99.9% of the vials had achieved the $L_{dry}$ condition following the UA. The three replicates of the normal operation runs had comparable results with an endpoint in between 21.48 and 21.55 h of drying. Whereas, the midpoint of $P_{ratio}$ was located in between 20.78 and 21.68 h and the offset between 22.73 and 23.77 h (see Table 3).

As expected, the endpoints of the disturbed verification runs are less precisely located. It appeared that there exists a strong correlation with the relative extend of the applied disturbances. Nevertheless, the location of the endpoint predictions according to the UA are analogous to the normal operation runs as they are situated between the midpoint and the offset of the $P_{ratio}$ curve. The relative difference between the UA prediction and the $P_{ratio}$ midpoint varied between 0.58 and 5.64% in time. In the final step after secondary drying, all vials were visually checked for defects such as collapse and meltback. No defects were noticed for vials coming from both the undisturbed and disturbed process, therefore indicating good performance of the controller.

## 4. Discussion and Perspectives

The observed $T_{i}$ values are very close (<1 ${}^{\circ }$C) to the predicted $T_{i}$ values. Moreover, no defects are observed after secondary drying. All of this indicates accurate model prediction for both the edge and centre vials and a competent real-time optimisation and control strategy to supervise the primary drying phase. Also for the disturbed verification runs, the observed model outcomes are quite close (<1.5 ${}^{\circ }$C) the the predictions. The proposed optimisation strategy kept the 99.9% upper percentile of $T_{i}$ almost constantly under $T_{c}$, i.e., equal to a RoF of 0.1. Hence, the primary drying process can be operated more efficiently by reducing the time needed to achieve the primary drying endpoint. Moreover, the nominal $L_{dried}$ and $T_{i}$ predictions were corrected after each horizon, which is especially useful when unexpected process disturbances occur. The ability of the machine capability space to move in both directions (i.e., changing $P_{c}$ and $T_{s}$) resulted in adequate responses to the artificially introduced disturbances.

In the case of the 99.9% upper percentile of $T_{i}$ surpassing the $T_{c}$ under severe process disturbances, the excursion time was kept as brief as possible. Nonetheless, this excursion time could be limited further by reducing the time resolution of the model-based controller. This would allow the controller to detected and prepare for disturbances more quickly. However, this would also lead to an increased computational load. Another strategy would be upgrading the cooling/heating or vacuum system of the freeze-dryer resulting in a larger operational space and thus more ample room for corrections.

The endpoint of primary drying evaluated with thermocouples was perceived earlier then the UA prediction. However, it should be noted that the former are not the most accurate method to determine the end of primary drying since they are based on point measurements in a single vial [20]. Moreover, the primary drying model assumes a flat sublimation plane whereas in practice some curvature of the plane is noticeable. This can lead to inaccurate model predictions of the endpoint. Hence, the endpoint by the proposed strategy was based on both the model and comparative pressure measurement. The $P_{ratio}$ proved to be corresponding more with model predictions then the thermocouples. The offset of $P_{ratio}$ was chosen as the start for secondary drying as this posses the least risks towards prematurely ending primary drying. However, near the end of primary drying some desorption of the dried layer could already start, delaying the offset of $P_{ratio}$ [12]. The midpoint of the $P_{ratio}$ curve was more comparable to the UA model predictions which is similar to the observations of Patel et al. [17].

A critical aspect of this work was to reduce the computational load of the real-time calculations. To solve this some practicalities such as sub-sampling, empirical fitting of the $T_{i}$ distribution and refining the prediction grid with a surface response model were introduced. This results in more efficient calculations while increasing the resolution of the dynamic design space. It should however be taken into account that this approach uses multiple successive fits, which makes that only an approximation of $T_{i}$, $L_{dried}$ and ${\dot{m}}_{sub}$ could be made.

Because the process is steered towards the edge of failure without crossing it, it appeared that the proposed optimisation and control strategy is very dependent on the model parameter values. The accuracy of the predicted $R_{p}$ and its variation is very important as this indirectly acts as a hard constraint to the operation of the process. Although the experimental fit of $R_{p}$ using regression Equation (6) described the data quite well, there exists a slight overprediction at low $L_{dried}$ (see Figure 3). However, this is not problematic as it will lead to more conservative control settings and therefore no extra risk on failure is created. Yet, Equation (6) is not capable of describing all possible $R_{p}$ curves perfectly [26]. More detailed equations describing this relation could be included in the future. Also note that the current strategy could further be improved if a tunable diode laser absorption spectroscopy (TDLAS) system would be present. With TDLAS, the vapour flow between the drying chamber and condenser can be measured in detail. Hence, the predictions of ${\dot{m}}_{sub}$ can be corrected and therefore more accurate real-time estimates of the $K_{v}$ and $R_{p}$ parameters can be made.

## 5. Conclusions

Recently new methodologies are being proposed to optimize batch freeze-drying by implementing dynamic settings during primary drying [4]. When a dynamic design space approach is used, the risk of collapse, choked flow and other failures can be estimated while optimising the processing time [8,20]. In this work a model-based supervisory optimisation and control strategy is proposed that takes into account model output uncertainties to maximise the sublimation rate ${\dot{m}}_{sub}$ of the primary drying phase. This is done while remaining below a predefined risk of failure (RoF), as determined by the critical sublimation interface temperature $T_{c}$, and while avoiding chocked flow conditions. Moreover, model uncertainties are updated in real time using common process measurements which allows for more aggressive operation when variations in the measurements are low and more conservative operation when required.

Multiple experimental verification runs, with and without induced process disturbance, were performed on a batch freeze-dryer with a predefined RoF of 0.1%. During these experiments the manipulated variables $T_{s}$ and $P_{c}$ were continuously optimised according to changing design space, which resulted in a dynamic primary drying trajectory. It was shown that the measured product temperatures were consistently located within the uncertainty range as predicted by the model. Moreover, the end of primary drying was also predicted correctly which resulted in compliant products with acceptable cake appearance. As such, it should be concluded that the proposed optimisation and control strategy can be successfully used to reduce processing times and operational costs of batch freeze-drying.

## Figures and Tables

**Table 1:** Sources of uncertainty in the primary drying model together with the methods used to take into account their variations.

Description	Symbol	Unit	Variation Term
*Parameters*			
Inner vial radius	$r_{i}$	m	standard deviation
Outer vial radius	$r_{o}$	m	standard deviation
Heat transfer coefficient	$K_{v}$	J/m${}^{2}$s·K	relative standard deviation
Dry layer resistance	$R_{p}$	m/s	standard deviation
Filling volume	$V_{fill}$	m${}^{3}$	standard deviation
*Variables*			
Shelf temperature	$T_{s}$	K	iterative determination
Chamber pressure	$P_{c}$	Pa	iterative determination
Dried layer thickness	$L_{dried}$	m	error propagation

**Table 2:** Nominal values of the parameters and constants for the proposed model-based optimisation and control strategy.

Description	Symbol	Numerical Value	Unit
Conversion factor a	*a*	88,9200	-
Conversion factor b	*b*	1.02	-
Inner vial radius	$r_{i}$	0.0110	m
Inner vial radius standard variation	$\sigma_{r_{i}}$	5.44 × 10^−5^	m
Outer vial radius	$r_{o}$	0.0120	m
Outer vial radius standard variation	$\sigma_{r_{o}}$	5.44 × 10^−5^	m
Vial neck radius	$r_{n}$	0.00630	m
Condenser duct radius	$r_{d}$	0.0800	m
$P_{i}$ coefficient	$A_{Pi}$	9.550426	Pa
$P_{i}$ coefficient	$B_{Pi}$	5723.2658	K
$P_{i}$ coefficient	$C_{Pi}$	3.53068	1/K
$P_{i}$ coefficient	$D_{Pi}$	0.00728332	Pa
$\Delta H_{sub}$ coefficient	$A_{H_{sub}}$	4.68 × 10^4^	J/mol
$\Delta H_{sub}$ coefficient	$B_{H_{sub}}$	35.9	J/mol·K
$\Delta H_{sub}$ coefficient	$C_{H_{sub}}$	0.0741	J/mol·K${}^{2}$
$\Delta H_{sub}$ coefficient	$D_{H_{sub}}$	542	J/mol
$\Delta H_{sub}$ coefficient	$E_{H_{sub}}$	124	K${}^{2}$
$K_{v}$ coefficient for edge vials	$\alpha_{e}$	−1.14	J/m${}^{2}$s·K
$K_{v}$ coefficient for centre vials	$\alpha_{c}$	3.46	J/m${}^{2}$s·K
$K_{v}$ coefficient for edge vials	$\beta_{e}$	4.46	J/m${}^{2}$s·KPa
$K_{v}$ coefficient for centre vials	$\beta_{c}$	1.93	J/m${}^{2}$s·KPa
$K_{v}$ coefficient for edge vials	$\gamma_{e}$	0.0757	1/Pa
$K_{v}$ coefficient for centre vials	$\gamma_{c}$	0.0292	1/Pa
$K_{v}$ relative standard deviation	$RSD_{K_{v}}$	0.0761	-
$R_{p}$ coefficient	$R_{p0}$	1.51 × 10^4^	m/s
$R_{p}$ coefficient	$A_{Rp}$	9.68 × 10^7^	1/s
$R_{p}$ coefficient	$B_{Rp}$	968	1/m
$R_{p}$ standard deviation	$\sigma_{R_{p}}$	1.10 × 10^4^	m/s
Critical temperature	$T_{c}$	238.9	K
Filling volume	$V_{fill}$	3.00 × 10^-6^	m${}^{3}$
Filling volume standard deviation	$\sigma_{V_{fill}}$	1.29 × 10^-8^	m${}^{3}$
Density of ice	$\rho_{ice}$	919.4	kg/m${}^{3}$
Density of solution	$\rho_{sol}$	1000	kg/m${}^{3}$
Porosity of dried layer	ϵ	0.97	-
Molecular weight of water	*M*	0.018015	kg/mol
Latent heat of sublimation	$\Delta H_{sub}$	51,136.82	J/mol
Thermal conductivity of ice	$\lambda_{ice}$	2.45	W/mK
Number of samples	$n_{sample}$	10,000	-
Time resolution primary drying prediction	Δt	60	s
Width of the prediction horizon	$\Delta t_{hor}$	120	s
Width of the $P_{c}$ machine capability space	$MCP_{Pc}$	1	Pa
Risk of Failure	RoF	0.1	%

**Table 3:** Summary of the end of primary drying verification for the six experimental runs.

Verification Experiment	$P_{\mathrm{ratio}}$ (h)		UA end (h)	Relative Difference Midpoint and UA (%)
	Midpoint	Offset		
Normal operation 1	20.78	22.73	21.55	3.57
Normal operation 2	20.96	23.99	21.48	2.42
Normal operation 3	21.68	23.77	21.49	0.88
Disturbances 1	22.22	23.87	22.35	0.58
Disturbances 2	21.38	23.32	22.37	4.43
Disturbances 3	22.08	24.52	23.45	5.64
